# The association of *FSCN1* (rs852479, rs1640233) and *HOTAIR* (rs920778) polymorphisms with the risk of breast cancer in Egyptian women

**DOI:** 10.1007/s11033-024-09459-9

**Published:** 2024-04-08

**Authors:** Eman Reda Galal, Dina A. Abdelhakam, Lamiaa Khalaf Ahmed, Yasmine Elhusseny, Sherif El Prince Sayed, Noha H. Eltaweel

**Affiliations:** 1 Biochemistry and Molecular Biology Department, Faculty of Pharmacy (Girls), Al-Azhar University, Cairo, Egypt; 2 Department of Clinical Pathology, Faculty of Medicine, Ain Shams University, Cairo, Egypt; 3 Medical Biochemistry and Molecular Biology Department, School of Medicine, Newgiza University, Giza, Egypt; 4 Department of General Surgery, Faculty of Medicine, Beni-Suef University, Beni Suef, Egypt; 5 Medical Molecular Genetics Department, Human Genetics and Genome Project Institute, National Research Centre, Cairo, Egypt

**Keywords:** Breast cancer, *FSCN1*, Gene polymorphism, *HOTAIR*

## Abstract

**Background:**

Breast cancer (BC) is one of the most prevalent cancers that contribute to mortality among women worldwide. Despite contradictory findings, considerable evidence suggests that single nucleotide polymorphisms (SNPs) in the *FSCN1* and *HOTAIR* genes may have a causative impact on the development of BC. This case–control study was conducted to evaluate the association of genotype frequency in *FSCN1* rs852479, rs1640233, and *HOTAIR* rs920778 with susceptibility and prognosis of BC, as well as the impact of clinical stages and hormonal features.

**Methods and results:**

*FSCN1* (rs852479, rs1640233) and *HOTAIR* (rs920778) were genotyped using TaqMan real-time PCR assay in 200 BC patients and 200 cancer-free controls, all representing Egyptian women. Genotypic analyses in association with clinicopathological factors and disease risk were assessed. As a result, a significant association with BC risk was observed for CC genotype frequency of *FSCN1* rs852479 A > C (OR = 0.395, 95% CI 0.204–0.76, *p*-*value* = 0.005). However, no significant correlation was detected between the *FSCN1* rs1640233 C > T and *HOTAIR* rs920778 C > T polymorphic variants and susceptibility to BC. Interestingly, CC genotype of *FSCN1* rs1640233 was more likely to progress tumor size and lymph node invasion in BC cases (*p*-*value* = 0.04 and 0.02, respectively). Moreover, it was revealed that there was a non-significant correlation between the haplotype distributions of *FSCN1* rs852479 and rs1640233 and the probability of BC.

**Conclusions:**

Based on the sample size and genetic characteristics of the subjects involved in the present study, our findings indicated that *FSCN1* rs852479 may contribute to BC susceptibility in a sample of the Egyptian population.

## Introduction

Breast cancer (BC) is the most prevalent malignancy in women worldwide. According to its death rate, it is considered the second most frequent cause of cancer mortality among women [1]. Globally, there were 2.3 million women diagnosed with BC and 685,000 deaths from this disease in 2020 [2]. The incidence and mortality rates of BC vary according to the region [3]. An estimated 313,510 new instances of invasive BC in women and 611,720 cancer-related deaths will occur in the United States in 2024 [4]. In Egypt, more than 22,000 new cases of cancer are diagnosed each year, making it the main cause of cancer-related mortality among Egyptian women [5]. The ratio of mortality/incidence rate of BC cases in Egypt was approximately double the ratio (41%) when compared with developed countries (23%) [6]. Many variables are associated with the risk of BC, including age, environmental, gynecological, and genetic factors [7].

Polymorphisms of a DNA sequence caused by a single nucleotide variation in humans are known as single-nucleotide polymorphisms (SNPs), which are the most prevalent types of genetic variations in the human genome. SNPs in genes can potentially alter the protein structure or affect the expression level of the gene product [8, 9], which in turn changes disease susceptibility, affecting tumorigenesis and cancer progression as well as drug resistance [10, 11]. Certain genetic polymorphisms can predict an individual’s susceptibility to BC and also influence disease management and progression [12].

Fascin-1 (*FSCN1*) is a 55-kDa actin-bundling protein coded by a gene located on chromosome 7p22.1 with about 13.84 kb in length and includes five exons. Human *FSCN1* is thought to be involved in the assembly of actin filament bundles found in lamellipodia, filopodia, microspikes, and stress fibers [13, 14]. *FSCN1* is abundantly expressed in many types of normal cells, including neurons, endothelial cells, glial cells, mesenchymal, and antigen-presenting dendritic cells, and is low or absent in normal epithelial cells [15]. Based on the occurrence of *FSCN1* in different organs, it is predictably participating in more biological functions in the human body [16]. In contrast to normal tissues, increased *FSCN1* expression has been associated with several types of malignancies, including lung, colon, breast, ovary, and oral squamous cell carcinoma [17–21]. As an oncogene, *FSCN1* can influence mitochondrial remodeling in cancerous cells, in addition to promoting invasion, tumor migration, metastatic colonization, cancer cell self-renewal, and drug resistance. In BC, *FSCN1* is crucial for predicting aggressive tumor behavior, especially in advanced stages [22]. Recent evidence suggests that aberrant STAT3 signaling accelerates the growth of breast tumors by downregulating the expression of downstream target genes that regulate angiogenesis, such as hypoxia-inducible factor-1 (HIF-1) and nuclear factor-kappaB (NF-κB), and by binding to the promoter of the *FSCN1* gene, triggering its expression [23]. Upregulation of FSCN1 enhances the severity and prognosis of human BC and can serve as a diagnostic marker to differentiate triple-negative subtypes of BC from other types of the disease [14]. Interestingly, different SNPs have been reported in *FSCN1* to modulate the risk of BC development [24].

*HOX* transcript antisense RNA (*HOTAIR*) is a transcript that originates from the antisense strand of the *HOXC* gene cluster with an approximate length of 2.2 kb. The human *HOTAIR* gene is found between *HOXC11* and *HOXC12* genes on the long arm of chromosome 12q13.13 [25]. It is an example of an oncogenic long noncoding RNA (lncRNA), which has emerged as a master regulator of cancer [12]. The *HOTAIR* gene controls several cellular and biochemical processes to promote the proliferation, invasion, survival, drug resistance, and prognosis of various tumors. Some reports indicate that polymorphisms of the *HOTAIR* gene are associated with a variety of cancers, including breast [25], pancreatic [26], gastric [27], thyroid [28], and colorectal cancers [29]. *HOTAIR* gene expression in BC cells is modulated by numerous epigenetic and transcriptional mechanisms [25]. Several SNPs, located in the intronic region of the *HOTAIR* gene, have been reported to regulate its expression level [30–32]. These SNPs are expected to be related to the occurrence, progression, recurrence, and metastasis of BC and serve as a novel therapeutic target for the disease [33, 34].

Recently, the relationship of *FSCN1* and *HOTAIR* polymorphisms with breast tumor development has been investigated [34–36]. However, some conclusions are still controversial and require further analysis to fully understand the relationship between these genes’ polymorphisms and BC risk. Therefore, this study was conducted to elucidate the association between *FSCN1* rs852479, rs1640233, and *HOTAIR* (rs920778) with the risk or prognosis of BC concerning several clinicopathological variables in the Egyptian population.

## Subjects and methods

### Study subjects

This study enrolled 200 Egyptian women with BC (cases) and 200 healthy women without BC (controls), matched by age and comparable socioeconomic factors. All participants were recruited from Beni-Suef University Hospital in the period between 2021 and 2023.

The study protocol was approved by the Ethics Committee of the Faculty of Pharmacy (Girls), Al-Azhar University (REC number: 436), and all study procedures were conducted in accordance with the Declaration of Helsinki. All study participants provided fully informed written consent at the time of study entry.

All samples underwent genotyping for three SNPs: *FSCN1* rs852479, rs1640233, and *HOTAIR* rs920778 to evaluate the association between gene polymorphisms and BC risk. Clinical examinations were detected to assess the impact of polymorphisms on BC patients based on menopausal status, tumor size, lymph node invasion, and histological grade. Additionally, BC prognostic biomarkers, including estrogen receptor (ER), progesterone receptor (PR), and human epidermal growth factor receptor 2 (HER2), were investigated.

### DNA extraction

A peripheral blood sample (3 ml) was withdrawn from all study participants under complete aseptic conditions. Genomic DNA was extracted from blood samples using the salting‐out method [37]. The concentration and quality of the DNA were checked by measuring the absorbance at 260 and 280 nm using a UV spectrophotometer, NanoDrop 2000 (Thermo-Fisher Scientific, Wilmington, USA). Pure preparations of DNA have OD260/OD280 values of 1.7–2.0. The extracted DNA concentration ranged from 50 to 100 ng DNA/μl. The extracted DNA samples were maintained at a temperature of − 20 °C until the genotyping procedure.

### Polymorphisms genotyping

Genotyping of *FSCN1* rs852479, rs1640233, and *HOTAIR* rs920778 was done by TaqMan real‐time PCR method using the pre-designed assays for allelic discrimination, containing specific TaqMan probes with fluorescent dyes for each allele. The total PCR volume was 20 µl, containing 5 μl DNA, 10 μl TaqMan Universal PCR Master Mix, 0.05 μl (40×) Assay Mix, and 4.5 μl RNase‐free water. The PCR reaction conditions were the same for the three SNPs, with a pre-denaturation cycle at 95 °C for 10 min, followed by 45 cycles of 95 °C denaturation for 10 s, 60 °C annealing for 30 s, and final extension at 72 °C for 30 s. For genotyping quality control, deionized water was used to replace template DNA as a negative control. The PCR results (changing fluorescence level) were analyzed using the provided software.

### Sample size and statistical analysis

The sample size was calculated using G*Power software version 3.1.9.7 for power analysis and sample size [38]. A total sample size of 400 was required, 200 in each group, with a power of 80% and a significance level of 5%. SPSS 22.0 software package (SPSS Inc., Chicago, IL, USA) was utilized for statistical analysis. Categorical variables are expressed as frequencies and percentages, while continuous variables are given as mean ± standard deviation (SD). Differences in clinical characteristics were compared between patients and healthy control groups using independent-sample t-tests (continuous variables) and chi-square tests (categorical variables). Hardy–Weinberg equilibrium (HWE) analysis was performed for each SNP assay. The chi-square (χ^2^) test was used to test differences between the two groups for each SNP genotype and allele. Allele frequencies were calculated with the gene counting method. The most common genotypes were selected as the reference. Odds ratios (OR) were calculated with a 95% confidence interval (CI) to estimate the degree of the association between genotypes and the risk of BC. SNPStats (https://www.snpstats.net/) was used to perform haplotype analysis test for linkage disequilibrium (LD) [39]. The significance level was set at a *p-value* < 0.05.

## Results

### General demographic and clinicopathological characteristics of the studied groups

The demographic and clinicopathological features of BC patients and controls in this study are shown in Table 1. The mean age of the controls at the time of enrollment was not significantly different from that of the BC cases (48.39 vs. 49.5 years, respectively, *p-value* = 0.394). Regarding BC cases, 47% were premenopausal, and 53% were postmenopausal. In respect to ER, 126 cases (63%) tested positive, and 125 cases (62.5%) tested positive for PR, while 45 (22.5%) of the cases were positive for HER2. Referring to the Nottingham prognostic index (NPI), the percentage of BC patients with T 1, 2, 3, and 4 was 9, 37.5, 28.5, and 25%, respectively, and N 0, 1, 2, and 3 emerged in 28, 43.5, 19, and 9.5% of patients, respectively. Concerning the histology grades of BC, 9 patients were classified as grade I (4.5%), 141 as grade II (70.5%), and 50 as grade III (25%).

**Table 1:** General demographic and clinical characteristics of the studied groups

	Patients group N = 200	Controls group N = 200	*p-value*
Age, years: (Mean ± S.D.)	49.5 ± 13.8	48.39 ± 13.23	0.394
Menopausal			
Pre	94 (47%)	–	–
Post	106 (53%)		
ER status			
Negative	74 (37%)	–	–
Positive	126 (63%)		
PR status			
Negative	75 (37.5%)	–	–
Positive	125 (62.6%)		
HER2 status			
Negative	155 (77.5%)	–	–
Positive	45 (22.5%)		
Tumor size			
T1	18 (9%)	–	–
T2	75 (37.5%)		
T3	57 (28.5%)		
T4	50 (25%)		
Lymph-node			
n0	56 (28%)	–	–
n1	87 (43.5%)		
n2	38 (19%)		
n3	19 (9.5%)		
Histological grade			
I	9 (4.5%)	–	–
II	141 (70.5%)		
III	50 (25%)		

Comparisons were carried out by independent sample *t*-test

Data are expressed as N (%): number of subjects (%) and means ± SDs; otherwise (–): not available

*p-value* < 0.05 was statistically significant

*ER* estrogen receptor, *PR* progesterone receptor, *HER2* human epidermal growth factor receptor 2

### Distribution frequencies of genotypes and alleles in BC patients and controls

**Fig. 1:**
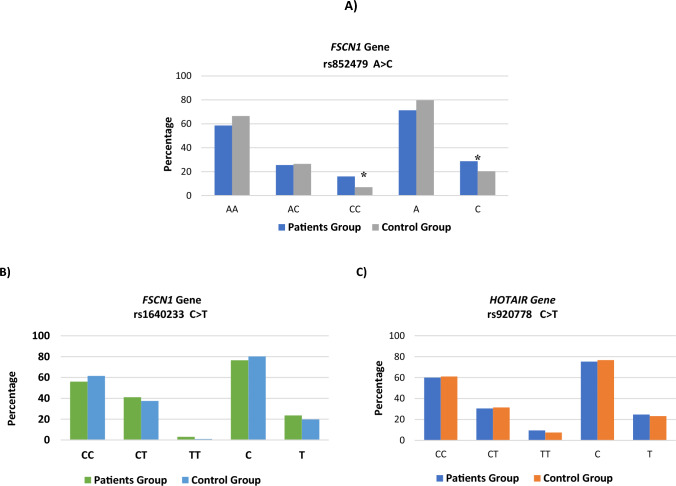
SNPs genotypes and allele frequencies of *FSCN1* and *HOTAIR* genes in different studied groups **A** Frequency of *FSCN1* gene rs852479, **B** *FSCN1* gene rs1640233, and **C** *HOTAIR* gene rs920778 genotypes and alleles among studied groups

The distribution patterns of *FSCN1* rs852479 and rs1640233, and *HOTAIR* rs920778 genotypes for all subjects are shown in Table 2 and Fig. 1. In the healthy controls and cases groups, all genotypic frequencies were in HWE (*p-value* > 0.05). Genotype analysis of *FSCN1* polymorphism in both controls and cases revealed that most of those with rs852479 SNP were homozygous for the AA genotype, while with rs1640233 SNP, most of them were homozygous for CC genotype. Likewise, for *HOTAIR* rs920778 SNP, most of the controls and cases were homozygous for CC genotype (Table 2).

**Table 2:** Genotype distribution and allele frequency between BC patients and healthy control group

SNP_ID	N (%) of Studied groups		*Pearson* *χ*^2^ value	OR (95% CI)	*p-value* (Sig.2)
	Patients group N = 200	Controls group N = 200			
*FSCN1 gene: rs852479 (A > C)*					
Genotypes			Ref		
AA	117 (58.5%)	133 (66.5%)			
AC	51 (25.5%)	53 (26.5%)	0.52	1.053 (0.67–1.64)	0.82
CC	32 (16%)	14 (7%)	7.95	0.395 (0.204–0.76)	0.005*
HWE	0.29	0.23			
Alleles			Ref		
A	285 (71.25%)	319 (79.75%)			
C	115 (28.75%)	81 (20.25%)	7.3	0.63 (0.46–0.88)	0.01*
*FSCN1 gene: rs1640233 (C > T)*					
Genotypes			Ref		
CC	112 (56%)	123 (61.5%)			
CT	82 (41%)	75 (37.5%)	0.514	0.863 (0.578–1.29)	0.474
TT	6 (3%)	2 (1%)	2.041	0.327 (0.065–1.632)	0.153
HWE	0.24	0.20			
Alleles			Ref		
C	306 (76.5%)	321 (80.25%)			
T	94 (23.5%)	79 (19.75%)	0.247	0.94 (0.66–1.34)	0.198
*HOTAIR gene: rs920778 (C > T)*					
Genotypes			Ref		
CC	120 (60%)	122 (61%)			
CT	61 (30.5%)	63 (31.5%)	0.047	1.04 (0.68–1.60)	0.829
TT	19 (9.5%)	15 (7.5%)	0.514	0.77 (0.38–1.56)	0.47
HWE	0.25	0.23			
Alleles			Ref		
C	301 (75.25%)	307 (76.75%)			
T	99 (24.75%)	93 (23.25%)	0.24	0.92 (0.66–1.27)	0.618

Bold values represent the statistically significant results (*p*-value < 0.05)

Comparisons were carried out by Chi-square (χ^2^) test

Data are expressed as N (%): number of subjects (%)

*p-value*: Sig. (two-tailed) * Significant; *p-value* < 0.05

*Ref* reference, *SNP* single nucleotide polymorphism, *HWE* Hardy–Weinberg equilibrium, *OR* odds ratio, *CI* confidence interval

According to the logistic regression analysis for each genetic polymorphism in BC patients and controls as given in Table 2, we observed that women with CC genotype frequency of *FSCN1* rs852479 A > C have a significantly high incidence of developing BC when compared with AC genotype (CC vs. AC, OR = 0.395; 95% CI 0.204–0.76, *p-value* = 0.005; OR = 1.053, 95% CI 0.67–1.64, *p-value* = 0.82, respectively). In addition, those with the C allele of the *FSCN1* rs852479 polymorphism were more likely than those with the A allele to develop BC (C allele OR = 0.63; 95% CI 0.46–0.88, *p-value* = 0.01) (Table 2; Fig. 1A). Regarding *FSCN1* rs1640233 C > T and *HOTAIR* rs920778 C > T polymorphism, differences in all genotypes were not significant for BC patients compared with healthy controls (Table 2; Fig. 1B, C).

### The association between genotypes and clinicopathological features of BC patients

In this study, the possible relationship between some clinicopathological parameters of patients with BC and the distribution of SNP genotypes was explored (Table 3). Regarding the clinical characteristics, only the *FSCN1* rs1640233 polymorphism of the CC genotype was significantly associated with developing tumor size and lymph node involvement among BC cases (*p-value* = 0.04 and 0.02, respectively). Otherwise, no significant differences were found in the frequencies of *FSCN1* rs852479 and *HOTAIR* rs920778 genotypes in the patients’ group based on all evaluated features (*p-value* > 0.05).

**Table 3:** Statistical Comparison of the demographic and clinical features with genotypes distribution of gene polymorphisms in patients group

	*FSCN1* gene rs852479A > C Genotype frequency (%)			*p-value*	*FSCN1* gene rs1640233 C > T Genotype frequency (%)			*p-value*	*HOTAIR gene* rs920778 C > T Genotype frequency (%)			*p-value*
	AA N = 117	AC N = 51	CC N = 32		CC N = 112	CT N = 82	TT N = 6		CC N = 120	CT N = 61	TT N = 19	
Age, years: (Mean ± S.D.)	49.8 ± 13.8	49.3 ± 14.7	52.1 ± 11.5	0.63	49.9 ± 14.13	49.9 ± 14.21	49.1 ± 14.4	0.68	48.2 ± 12.7	51.1 ± 14.3	50.4 ± 15.7	0.61
Menopausal												
Pre	53 (45.3%)	25 (49%)	17 (53.12%)	0.71	52 (46.4%)	41 (50%)	2 (33.3%)	0.69	56 (46.7%)	32 (52.5%)	7 (36.8%)	0.476
Post	64 (54.7%)	26 (51%)	15 (46.88%)		60 (53.6%)	41 (50%)	4 (66.7%)		64 (53.3%)	29 (47.5%)	12 (63.2%)	
ER status												
Negative	47 (40.17%)	18 (35.3%)	9 (28.12%)	0.43	47 (42%)	26 (31.7%)	1 (16.6%)	0.19	51 (42.5%)	18 (29.5%)	5 (26.3%)	0.14
Positive	70 (59.83%)	33 (64.7%)	23 (71.88%)		65 (58%)	56 (68.3%)	5 (83.4%)		69 (57.5%)	43 (70.5%)	14 (73.7%)	
PR status												
Negative	43 (36.75%)	20 (39.2%)	12 (37.5%)	0.90	42 (37.5%)	30 (36.6%)	3 (50%)	0.80	40 (33.3%)	27 (44.3%)	8 (42.1%)	0.328
Positive	74 (63.25%)	31 (60.8%)	20 (62.5%)		70 (62.5%)	52 (63.4%)	3 (50%)		80 (66.7%)	34 (55.7%)	11 (57.9%)	
HER2 status												
Negative	89 (70.06%)	40 (78.4%)	26 (81.25%)	0.81	82 (73.2%)	67 (81.7%)	6 (100%)	0.153	91 (75.8%)	50 (82%)	14 (73.7%)	0.596
Positive	28 (23.94%)	11 (21.6%)	6 (18.75%)		30 (26.8%)	15 (18.3%)	0 (0%)		29 (24.2%)	11 (18%)	5 (26.3%)	
Tumor size												
T1	8 (6.8%)	6 (11.8%)	4 (12.5%)	0.38	9 (8%)	7 (8.5%)	2 (33.3%)	0.04*	9 (7.5%)	6 (9.8%)	3 (15.8%)	0.913
T2	44 (37.6%)	15 (29.4%)	16 (50%)		34 (30.4%)	40 (48.8%)	1 (16.7%)		45 (37.5%)	23 (37.7%)	7 (36.8%)	
T3	33 (28.2%)	18 (35.3%)	6 (18.75%)		36 (32.1%)	20 (24.4%)	1 (16.7%)		37 (30.8%)	17 (27.9%)	3 (15.8%)	
T4	32 (27.4%)	12 (23.5%)	6 (18.75%)		33 (29.5%)	15 (18.3%)	2 (33.3%)		29 (24.2%)	15 (24.6%)	6 (31.6%)	
Lymph-node												
n0	29 (24.8%)	16 (31.4%)	11 (34.3%)	0.44	21 (18.8%)	33 (40.2%)	2 (33.3%)	0.02*	31 (25.8%)	20 (32.8%)	5 (26.3%)	0.185
n1	51 (43.6%)	21 (41.2%)	15 (46.9%)		50 (44.6%)	33 (40.2%)	4 (66.7%)		51 (42.5%)	29 (47.5%)	7 (36.85%)	
n2	23 (19.7%)	12 (23.5%)	3 (9.4%)		28 (25%)	10 (12.2%)	0 (0%)		27 (22.5%)	8 (13.1%)	3 (15.8%)	
n3	14 (11.9%)	2 (3.9%)	3 (9.4%)		13 (11.6%)	6 (7.32%)	0 (0%)		11 (9.2%)	4 (6.6%)	4 (21.05%)	
Histological grade												
I	4 (3.42%)	5 (9.8%)	0 (0%)	0.153	5 (4.5%)	3 (3.7%)	1 (16.7%)	0.61	7 (5.8%)	1 (1.6%)	1 (5.25%)	0.092
II	82 (70.08%)	37 (72.55%)	22 (68.75%)		80 (71.4%)	58 (70.7%)	3 (50%)		89 (74.2%)	41 (67.2%)	11 (57.9%)	
III	31 (26.5%)	9 (17.65%)	10 (31.25%)		27 (241%)	21 (25.6%)	2 (33.3%)		24 (20%)	19 (31.1%)	7 (36.85%)	

Bold values represent the statistically significant results (*p*-value < 0.05)

Data are expressed as N (%): number of subjects (%), and means ± SDs

*p-value*: Sig. (two-tailed) * Significant; *p-value* < 0.05

*ER* estrogen receptor, *PR* progesterone receptor, *HER2* human epidermal growth factor receptor 2

### The association of FSCN1 haplotype frequencies with BC in the studied groups

Association analysis between the risk of BC and haplotypes of *FSCN1* rs852479 and rs1640233 among BC cases and controls is summarized in Table 4. LD was estimated for the two SNPs (r^2^ = 0.65, D′ = 0.88), which is expected under linkage disequilibrium. The haplotypes’ distribution showed that the AC haplotype was the most frequent in both cases and controls (69.9 and 77.2%, respectively), while AT haplotype showed the lowest frequency among both groups. Overall, none of the considered haplotypes were significantly associated with the development of BC (*p-value* > 0.05).

**Table 4:** Association of *FSCN1* rs852479and rs1640233 haplotypes with BC

Haplotype	Patients group Frequency	Controls group Frequency	OR (95% CI)	*p-value*
rs852479–rs1640233 (r^2^ = 0.65, D′ = 0.88)				
AC	0.6993	0.7717	Ref	
CT	0.2218	0.1717	0.82 (0.55–1.24)	0.35
CC	0.0657	0.0308	0.50 (0.23–1.08)	0.077
AT	0.0132	0.0258	1.79 (0.59–5.39)	0.3

*p-value* < 0.05 was statistically significant

*Ref* reference, *OR* odds ratio, *CI* confidence interval

## Discussion

BC is a complicated and heterogeneous disease with a multifaceted etiology caused by a combination of genetic and lifestyle-related factors. Various studies suggest that SNP genotyping may contribute to risk assessment and guide BC management. In the present case–control study, we evaluated the frequency distributions of the *FSCN1* (rs852479, rs1640233) and *HOTAIR* (rs920778) SNPs and their associations with BC susceptibility in Egyptian women.

Regarding *FSCN1*, our findings revealed that women with CC genotype frequency of rs852479 C > A are significantly associated with a high risk of developing BC when compared with AC genotype (CC vs. AC, OR = 0.395, 95% CI 0.204–0.76, *p-value* = 0.005; OR = 1.053, 95% CI 0.67–1.64, *p-value* = 0.82; respectively). Furthermore, the *FSCN1* rs852479 C allele polymorphism is attributed to increased BC risk when compared with the frequency of A allele (C allele OR = 0.63; 95% CI 0.46–0.88, *p-value* = 0.01). In contrast, the rs1640233 SNP polymorphism of patients and controls did not differ significantly across all genotypes (*p-value* > 0.05).

Wang et al. [24] investigated the relationship between six SNPs of the *FSCN1* gene in a cohort of Han Chinese women. There were no significant variations detected in the genotypes’ frequency of the rs8772, rs3801004, rs2966447, rs852479, and rs1640233 polymorphisms between BC patients and the healthy control group [24]. Nevertheless, another study revealed that Egyptian females with the *FSCN1* rs3801004 C > G polymorphisms had a significantly higher risk of BC [36]. Liu et al. [40] suggest that there might be an association between *FSCN1* and the development of BC. They further confirmed the possible functional relevance of *FSCN1* expression in the development of Triple-Negative Breast Cancer (TNBC) because it was substantially higher in TNBC than in the non-TNBC subtype. Consequently, these results assist in elucidating the functional significance of *FSCN1* in the pathogenesis of TNBC and may provide perspectives on the mechanisms behind cancerous progression [40].

Concerning *HOTAIR* rs920778 C > T polymorphism, we reported no significant difference between the cases and control group (*p-value* > 0.05) for all genotypes and alleles. Our results are consistent with a recent study on the Egyptian population, which discovered that the rs920778 C > T polymorphism was not significantly related to BC progression [35]. According to prior research, the allelic frequencies of the *HOTAIR* gene (rs12826786, rs1899663, and rs4759314) were not statistically different between BC patients and cancer-free controls and were not likely to develop BC [41].

Contrary to our findings, it has been observed that there was a significant relationship between the rs920778 polymorphism and a high incidence of BC in women from Turkey [42], Iran [43], India [44], and China [30, 45]. Based on a meta-analysis of 4 studies with 4936 cases and 5214 healthy controls investigating the association of four *HOTAIR* SNPs with BC vulnerability, it was found that rs920778 polymorphism significantly lowered the risk of BC under heterozygous, homozygous, and recessive models among the West Asians, and increased BC risk under dominant and allele models within the East Asian population [34]. Furthermore, some reports have indicated that *HOTAIR* SNP rs920778 exhibits variable results in the same population but in distinct cancer types such as gastric [46] and breast [42], which suggests that there are variations in the polymorphism throughout different malignancies.

These disparities in the results could be caused by genetic diversity among ethnic populations resulting from different gene–gene and gene–environment interactions, or they could be the result of additional constraints associated with the number of cases and sampling techniques. As elucidation, the HapMap data (https://www.ncbi.nlm.nih.gov/snp/rs920778) indicates that there are notable variations in the allele frequency of the *HOTAIR* rs920778 polymorphism between various ethnic communities. Additionally, the assessment of HOTAIR expression in tumor samples could help in better recognition of the role of these polymorphisms in cancer progression, which ought to be investigated further [32].

Numerous investigations have been conducted on the relationship between gene polymorphisms involved in different cellular processes and the risk and clinicopathological aspects of BC. When we analyzed the clinical aspects of rs852479 and rs1640233 *FSCN1* and rs920778 *HOTAIR* genotypic frequencies among BC patients, we found that CC genotype of *FSCN1* rs1640233 was significantly associated with developed tumor size and lymph node invasion (*p-value* = 0.04 and 0.02; respectively). Besides, no statistically significant differences were identified in the frequencies of *FSCN1* rs852479 and *HOTAIR* rs920778 genotypes concerning all evaluated parameters (*p-value* > 0.05). Within the same context, other investigations suggested that there was no significant correlation between the clinicopathological aspects of BC patients and the *HOTAIR* rs920778 polymorphism [30]. Interestingly, Hassanzarei et al. [43] discovered that the frequencies of different *HOTAIR* genotypes in the Iranian population weren’t associated with any clinicopathological features except for rs920778, which was significantly related to ER status. Conversely, Bayram et al. [42] found that the CC genotype of *HOTAIR* rs920778 polymorphism was associated with advanced TNM classification, larger tumor size, poor histological grade, and the presence of distant metastasis in BC patients but was not related to other clinic-laboratory or hormonal parameters.

In a comparison of clinic-pathological aspects with *FSCN1* genotypes, Wang et al. [24] discovered that BC patients with the *FSCN1* rs852479 and rs1640233 were not statistically correlated to any clinical status of the tumor. Using immunohistochemistry, Min et al. [47] investigated *FSCN1* expression in a microarray of 194 samples from patients with invasive breast cancer. Findings suggested a strong correlation between the expression of *FSCN1* and some clinicopathological characteristics, such as high histological grade, tumor necrosis, and status of ER- and PR-negativity. They further found that *FSCN1* expression was significantly associated with BC survival, especially in patients with advanced-stage BC [47]. Moreover, in Chinese and African-American women, *FSCN1* expression is suggested to be associated with TNBC and also linked to more severe clinical aspects and negative hormone receptors [48, 49]. Haplotype analyses may provide evidence about the genetic involvement in disease incidence [50]. We examined the impacts of different haplotype combinations of two *FSCN1* SNPs rs852479 and rs1640233 upon the risk of BC, and no significant relation between haplotypes and BC susceptibility was detected. Overall, as related to other functional polymorphisms in other genes, the effect of genetic polymorphisms of *FSCN1* and *HOTAIR* on predisposition to BC would be affected by additional factors in these genes or perhaps other genes, and the assessment should be customized on a population-specific criterion.

## Conclusions

The findings of this study suggest that *FSCN1* rs852479 C > A polymorphism is implicated in BC risk and development among Egyptian women. Furthermore, CC variant of *FSCN1* rs1640233 C > T has been found to be significantly associated with some BC prognostic factors, potentially worsening the prognosis for those carrying the polymorphism. Otherwise, no significant relationship between the *HOTAIR* rs920778 C > T polymorphism and BC risk in our patients was detected. To our knowledge, this is the first study regarding *FSCN1* rs852479 and rs1640233 polymorphisms and their association with BC susceptibility in Egyptian women. Further studies are needed to be conducted in larger patient cohorts to explore specific clinical and pathological characteristics as well as in patients from different populations.

## Data Availability

The data sets generated and/or analyzed over the course of the study are not publicly available but are available from the corresponding author upon reasonable request.

## Abbreviations

BC: Breast cancer
SNPs: Single nucleotide polymorphisms
*FSCN1*: Fascin-1
*HOTAIR*: *HOX* Transcript antisense RNA
TNBC: Triple-negative breast cancer
